# Palliative care and human rights in patient care: an Armenia case study

**DOI:** 10.1186/s40985-017-0062-7

**Published:** 2017-08-07

**Authors:** Gabriela Barros de Luca, Violeta Zopunyan, Naomi Burke-Shyne, Anahit Papikyan, Davit Amiryan

**Affiliations:** 1 Open Society Foundations, New York, USA; 2 Center for Rights Development, Yerevan, Armenia; 3 Open Society Foundations, Westminster, UK; 4 Open Society Foundations - Armenia, Yerevan, Armenia

**Keywords:** Armenia, Palliative care, Opioid, Human rights, Pain, Patient, Provider, Human rights in patient care

## Abstract

This paper examines palliative care within the human rights in patient care framework, which clarifies state obligations and addresses the rights of both patients and providers. In the context of palliative care, these rights extend beyond the right to health and include patient rights to freedom from torture, cruel and inhuman treatment, non-discrimination and equality, bodily integrity, privacy and confidentiality, information, and right to a remedy. They also encompass provider rights to decent working conditions, freedom of association, and due process. The paper then looks at a case study of Armenia, acknowledging how the government’s commitment to palliative care, combined with awareness raising and advocacy by human rights organizations, created an enabling environment for the realization of human rights in patient care in the context of palliative care.

## Background

Patient care is a crosscutting human rights issue and an important aspect of the right to health. As Cohen and Ezer explain, the concept of human rights in patient care is derived *from inherent human dignity and neutrally applies universal, legally recognized human rights principles, protecting both patients and providers*. It further recognizes the interrelation between patient and provider rights and focuses on responsibilities of the state [1].

In the delivery of palliative care within a complex health system, palliative care patients and providers may experience abuse of their rights. This paper will examine palliative care within the human rights in patient care framework, with consideration to the rights of palliative care patients and providers, and the state’s obligations to respect, protect, and fulfill human rights in patient care. It will then apply these principles to a concrete case study and consider how Armenia’s commitment to the development of palliative care has advanced human rights in patient care.

## Human rights in palliative care

Founded upon a firm belief in compassion and the dignity of the human person, palliative care aligns closely with the principles of human rights in patient care. Palliative care improves the quality of life of patients and is defined as the holistic management of physical, psychological, legal, and spiritual problems faced by patients with life-threatening illness and by their families. In terms of managing physical problems faced by patients, it provides relief from distressing symptoms including pain, shortness of breath, fatigue, constipation, nausea, loss of appetite, problems with sleep, and many other symptoms [2, 3].

The World Health Organization (WHO) indicates that only 14% of people who need palliative care worldwide currently receive it [4]. An important part of palliative care is addressing chronic and severe pain. Every year, tens of millions of people suffer from moderate to severe pain without access to appropriate medicine for pain, including 5.5 million terminal cancer patients, 1 million end-stage HIV/AIDS patients, and 800,000 patients suffering injuries caused by accidents or violence [5]. With the older population, one of the most rapidly growing age groups in the world [6], and the increasing prevalence of non-communicable diseases, [7] the need for palliative care is only expected to rise [8]. The Worldwide Palliative Care Alliance recommends that all governments integrate palliative care into their country’s health system alongside curative care [9]. The patient focus in palliative care means it can be delivered in a variety of settings, including in hospitals, outpatient clinics, residential hospices, nursing homes, community health centers, and patients’ homes.

The WHO currently lists 20 palliative care medications in its essential medicines list [10]. These medicines, which include morphine and other opioid analgesics, are indispensable for treating the various symptoms, particularly pain, and other symptoms associated with many life-limiting conditions [4]. The importance of ensuring access to pain medicines was also affirmed by the World Health Assembly in its first global resolution on palliative care in 2014, which acknowledges that palliative care is a core component of health systems and calls upon the WHO and Member States to ensure the availability of narcotic medicines for symptom management (also referred to as “controlled medicines,” which are those medicines that have manufacture, possession, or use regulated by a government) [11]. Morphine, in particular, is not protected by patent and can cost as little as a few US cents per unit [12]. Yet, approximately 80% of the world’s population still has no access to it [5]. This figure reflects gross inequalities in access to controlled medicines for pain treatment: 92% of the world’s morphine is consumed by 17% of the world’s population, primarily in North America, Oceania, and Western Europe [13].

International human rights law has long considered palliative care integral to the right to health and the availability of essential pain medicines as one of the right’s core components (see also [14, 15]). Palliative care is recognized in the UN Committee on Economic Social and Cultural Rights’ (CESCR) authoritative interpretation of the right to the highest attainable standard of health (General Comment 14), whereby it provides that states are obliged to respect the right to health by, inter alia, refraining from denying, or limiting equal access for all persons to preventive, curative, and palliative health services [16]. The CESCR also provides that states cannot, *under any circumstance whatsoever*, justify non-compliance with the obligation to provide access to essential medicines, as defined by the WHO essential medicines list [10, 16]. It has also affirmed the importance of providing care for chronically and terminally ill persons, “sparing them avoidable pain and enabling them to die with dignity” [16].

The Inter-American Convention on Protecting the Human Rights of Older Persons, adopted in June 2015, is the first treaty to explicitly articulate a right to palliative care. It draws on a number of rights to lay out a package of essential safeguards and guarantees that are critical to the right to palliative care. Under the Inter-American Convention, states are required to provide access to palliative care without discrimination, prevent unnecessary suffering and futile procedures, and enable older persons to expressly indicate in advance their will and instructions with regard to health care interventions, including palliative care [17].

This paper will discuss rights relevant to patients in the context of palliative care beyond the right to health; to freedom from torture and cruel, inhuman, and degrading treatment; [18–22] to non-discrimination and equality; [18, 20, 21, 23–26] to bodily integrity; [20, 24–27] to information; [18, 24–26] to privacy and confidentiality; [18, 21, 27] and to a remedy [18, 21, 24, 28]. Additionally, it will examine the rights relevant to palliative care providers, to decent working conditions, [23, 24] to freedom of association, [14, 18, 21], and to due process [18, 21, 24].

### Palliative care patients’ rights

International conventions speak specifically on the issue of the right to be free from torture and cruel, inhuman, and degrading treatment. Access to adequate pain treatment has been considered a requirement under this right. Two consecutive UN Special Rapporteurs on Torture and the UN Special Rapporteurs on Health have stated that the denial of access to controlled pain relief medicines, if it causes severe pain and suffering, may amount to cruel, inhuman, or degrading treatment or punishment: “The failure to ensure access to controlled medicines for the relief of pain and suffering threatens fundamental rights to health and to protection against cruel inhuman and degrading treatment… [Governments] have an obligation to take measures to protect people under their jurisdiction from inhuman and degrading treatment. Failure of governments to take reasonable measures to ensure accessibility of pain treatment, which leaves millions of people to suffer needlessly from severe and often prolonged pain, raises questions whether they have adequately discharged this obligation” [29–31].

The right to non-discrimination and equality, part of many human rights treaties, is also particularly relevant to palliative care patients. General Comment 14 provides that health facilities, goods, and services must be accessible to all, especially the most vulnerable or marginalized sections of the population, without discrimination on any of the prohibited grounds of “race, colour, sex, language, religion, political or other opinion, national or social origin, property, birth or other status” [16]. This means people living with HIV/AIDS, people who use drugs, sex workers, and ethnic minorities cannot be denied palliative treatment or care and must be provided with the same level of care, in the same conditions, as other patients [1].

The right to bodily integrity and the right to information are also relevant in the context of patient rights and palliative care and are often interconnected. The right to bodily integrity refers to a patient’s autonomy and self-determination over his/her own body, and deems any unconsented intrusion—physical or non-physical—to be a violation of this right, including medical treatment performed without a patient’s informed consent or ignoring patient wishes regarding treatment.

The right to information requires that, prior to consent, information be provided about the likely benefits and risks of proposed treatment and non-treatment [32]. In the context of palliative care, the right to information may include persons receiving all relevant information about their prognoses, treatment options, and the side effects of medication available to treat their conditions or symptoms.

Key international human rights instruments provide that medical information must be provided to the patient in an accessible and easily understood manner which is commensurate with cultural identity, level of education, and communication needs, and which respects the right to give and receive instructions in advance with regard to health care interventions. Importantly, persons receiving palliative care are also entitled to the right to privacy and confidentiality [17, 18, 23]. This means that patients have the right to have their health information and data kept confidential. Patients must always be given access to their own health information and be able to authorize the disclosure of such information [32].

The application of a human rights framework to patient care requires a range of measures, such as adequate laws and policies, documentation of abuses within healthcare service delivery, and—notably—legal remedies to address them [1]. When the state takes no effective action to address the violations of the human rights relevant to patient care, the patient’s right to a remedy is also violated (for more rights engaged in this context see [1, 32]).

### Palliative care providers’ rights

The human rights of palliative care providers (doctors, nurses, social workers, and professional caregivers) are inherently connected to the rights of patients. A framework of rights protecting providers means providers are more likely to be supported and motivated to provide palliative care in a manner that upholds the rights and dignity of patients and may mean greater state commitment to resourcing healthcare systems [1].

Providers of palliative care have the right to decent working conditions. They are entitled to enjoy just and favorable conditions which ensure fair wages, safe and healthy working conditions, and reasonable limitation of working hours. Examples of violations of this right include palliative care nurses being paid less than the national minimum wage, being required to work for excessive periods of time, or medical staff being exposed to high levels of radiation in the treatment of cancer patients [23].

Likewise, palliative care providers must have the right to freedom of association so that they may join professional associations where they can negotiate their working conditions, have access to continued training opportunities, and have the tools to challenge laws and practices that impose obstacles to their work [23]. It is equally important that palliative care providers have the right to due process if faced with any concerns related to their employment or medical practice [18]. For example, if a nurse facing disciplinary proceedings is unable to obtain access to all the evidence presented against her in advance of the hearing, or a doctor facing a medical negligence suit has still not been given a hearing date 5 years after commencement of the proceedings, both are situations that violate due process and may have a direct impact on whether or not the palliative care provider is able to continue providing care.

## Palliative care rights in Armenia

Armenia gained independence from the Soviet Union in 1990 but has largely preserved its centralized health care system—which guarantees free medical care and access to a comprehensive range of primary, secondary, and tertiary health care services for the entire population [33]. Armenia has also ratified and acceded to a number of key international human rights treaties, including the International Covenant on Economic, Social and Cultural Rights and the International Covenant on Civil and Political Rights (ICESCR), which protect the palliative care patients’ and providers’ rights, as discussed above [34]. This historical context creates both advantages and challenges to Armenia’s establishment and scale-up of palliative care.

Drug regulations are at the heart of problems with availability and accessibility of palliative care in Armenia, but many other human rights in patient care issues need urgent attention. Patients and their families do not have sufficient access to information about palliative care services and the option of palliative care, implicating the right to information. Ideally, palliative care should be a well-publicized service within communities. Patients with cancer should receive the information from their doctors so that palliative care would go hand in hand with definitive treatment and would be available for support at any stage of the illness. In fact, the physicians conceal the diagnosis from their patients in accordance with the legal provisions, which state that the disclosure of information about a patient’s illness or medical test results by medical personnel without any professional or official need is criminal. This signals that information deemed medically confidential may be disclosed only upon the request of the courts, the prosecutor’s office, authorities carrying out investigations, and other authorized entities in situations and according to procedures set by law. Right to confidentiality is also violated by the police officers, who collect information about patients, their proxies, and patients’ dosage of narcotic drugs from the doctors of policlinics without any legislative basis. Police control over the prescription and dispensing process is tight, invasive, and generates a sense of trepidation among oncologists and pharmacists [35]. At present, there exist a number of gaps preventing patients with long-term illnesses in Armenia from exercising their right to adequate pain treatment. Effective analgesics and other pain treatment medications do not reach these patients for a variety of reasons, including inadequate domestic legislation, a lack of oral opioids, a shortage of properly trained palliative care specialists, and low levels of awareness about pain treatment possibilities among patients and their relatives, implicating the right to freedom from torture and cruel, inhuman, and degrading treatment. These problems are compounded by widespread and exaggerated fears of addiction, particularly among law enforcement agencies and other state actors, who favor a highly restrictive approach to pain treatment medication.

In 2009, the Pain Control and Palliative Care Association, with support of international expertise and the Ministry of Health, commenced an assessment of the national need for palliative care. The assessment showed that 60–70% of the total annual cases of mortality in Armenia need palliative care—approximately 18,000 patients per year (there were 27,000–28,000 deaths per year in Armenia at the time of the assessment). This situation is exacerbated by the fact that a high percentage of cancer patients are diagnosed at advanced stages, about 46% of patients are diagnosed at stage three or four, when treatment options are limited and palliative care is critical. As the concept of holistic palliative care encompasses family needs, the assessment considered that, for every patient with a life-threatening illness, there are at least two family members requiring palliative care support [36].

Armenia also faces barriers to accessing opioid medicines for pain treatment. Although not explicitly required by law, opioid medicines are subject to an onerous prescription process requiring approval by the patient’s oncologist (in the case of cancer), the polyclinic’s chief and/or deputy chief doctor, chief nurse, general practitioner, and, in some cases, one or two other specialized doctors who work at the clinic. Armenian regulations also require that police maintain oversight of doctors prescribing and healthcare facilities storing opioids, sometimes requiring patients to return empty ampoules. Moreover, opioid medicines are available through only one specialized pharmacy in the capital, Yerevan. It is thus a time-intensive and bureaucratic process for patients and families seeking opioid medicines for pain treatment in the context of palliative care.

Since the 2009 assessment, Armenia has seen structural reforms, frameworks, and policies related to palliative care being implemented with some initial achievements in human rights in patient care. The following section will highlight the impact of reforms in terms of raising awareness of patients’ human rights and addressing pain treatment, and creating a protective, supportive, and enabling environment for healthcare workers in palliative care.

In 2011–2012, the Pain Control and Palliative Care Association at the Republican National Oncology Hospital in Yerevan, to provide trainings for oncologists in Armenia, established the first palliative care training center. Notwithstanding the center’s limited capacity and the fact that it is still developing a systematic approach to trainings, trained oncologists were observed to notably increase their daily pain medication prescriptions (on average from 60 mg of morphine per day per clinic to 250 mg of morphine per day per clinic—a more appropriate dosage based on international norms and standards) [37].

From 2011 to 2013, the Ministry of Health implemented palliative care pilot projects in four medical centers with support from the Global Fund to Fight AIDS, Tuberculosis and Malaria and the Open Society Institute Assistance Foundation Armenia. These pilot projects revealed the high levels of pain suffered by most patients seeking palliative care in the country (the mean admission pain scale score was considered to be high, requiring rapid intervention), which indicated the need for more training for healthcare workers and prompted further efforts to address barriers to pain treatment [38]. This was taken forward by national policies, Armenia’s 2012–2016 Palliative Care Concept Paper and its Action Plan for the Implementation of Palliative Care Services, which identified legal and practical problems impacting palliative care and, in particular, highlighted the right of patients to be free from degrading treatment (avoidable pain) [39]. In 2014, by signing the WHA resolution on “Strengthening of palliative care as a component of comprehensive care throughout the life course,” Armenia made a commitment to develop national policy, remove the restrictive legal procedures for prescribing opioids for patients with chronic life-long conditions, build capacity, and establish services in palliative care [11]. In December 2014, the Ministry of Health approved three policy documents on clinical guidelines for pain management, on standards for the provision of palliative care services, and on the professional qualification of doctors and nurses [40–42]. There are some promising developments underway regarding the development of palliative care. The government adopted the National Strategy for 2017–2019. The strategy provides provisions for developing services and improving regulations to prescribe opioids. Oral morphine was registered, yet is not available in the country.

Other gaps and barriers identified in the context of patient and provider rights in Armenia included the need to establish an independent body of medical professionals and ethicists mandated to resolve disputes between patients and providers [43]. The Armenian Ministry of Health has since established a national-level Medical Ethics Committee. At the time of writing this paper, the Medical Ethics Committee was not yet operational, still awaiting regulation defining the roles and responsibilities of committee members.

### Raising awareness of patients’ human rights and addressing pain treatment

The 2015 Human Rights Watch Report “All I Can Do Is Cry: Cancer and the Struggle for Palliative Care in Armenia” (HRW Report) presented a stark and shocking account of the lack of treatment for pain in Armenia. The report emphasized the suffering of thousands of patients with life-limiting illnesses, the restrictive government regulations on accessing strong pain medication, and challenges posed by the limited skills and knowledge of healthcare professionals in the area of pain treatment [35].

In parallel to the publication of that report, physicians, providers, patients and their families, and non-governmental organizations (NGOs) conducted a campaign called “Life without Pain” [44]. The campaign mobilized thousands of people through social media, face to face discussions, and meetings, emphasizing that access to oral opioids for pain relief is essential for quality palliative care and could transform the lives of tens of thousands of people in need of palliative care in Armenia. Campaign materials connected citizens with relevant NGOs and highlighted the connection between access to opioid pain medicines in palliative care with domestic and international human rights protections. The NGO Real World Real People, in cooperation with the Helsinki Citizens Assembly and the Center for Rights Development, documented the systemic gaps in accessing pain relief and offered legal aid to patients in palliative care lacking adequate pain relief. The publication of the HRW report and local advocacy coincided with the early implementation of a number of relevant government initiatives and encouraged the government to honor its commitments, which have since advanced access to palliative care and appropriate pain treatment.

Even with these structural changes and international guidance indicating oral morphine is the gold standard for combatting severe pain, access to opioid pain medicine remains a significant challenge in Armenia. National drug control policy continues to negatively impact upon the availability and accessibility of pain medication by imposing excessive administrative hurdles and requiring multiple consultations or approval thresholds for the prescription of opioid medicines for pain relief. The requirement to report opioid prescriptions to the police also continues to generate a sense of fear among doctors and pharmacists. Compounding this, oral morphine is not yet available in Armenia, meaning palliative care patients can access injectable morphine at best—despite the WHO’s recommendation that pain medication be delivered in oral form when possible [45]. However, following multi-stakeholder advocacy, the authors anticipate Armenia is on track to register oral morphine in 2017.

### Creating a protective, supportive, and enabling environment for healthcare workers

In late 2014, palliative care was formally recognized as a field of sub-specialization in medicine and a palliative care department was established in the Yerevan State Medical University—a first step towards integrating palliative care into the curricula of healthcare professionals more broadly. Most recently, in April 2015, the parliament adopted amendments to the Law on Medical Care and Services (the Medical Care Law) to include the definition of palliative care. The Medical Care Law explicitly sets out the state’s responsibility to meet specific standards on the implementation of palliative care services. Importantly, this regulatory framework creates the legal basis to allow both patients and providers to demand proper conditions for the implementation of palliative care services [46]. Additionally, Armenia’s National Strategy on Improving Child and Adolescent Health explicitly recognizes and defines pediatric palliative care [47].

The 2017–2020 National Strategy on Palliative Care will consolidate important developments in the provision of palliative care services, particularly from the perspective of healthcare workers. The strategy document focuses on building the capacity of doctors and nurses for palliative care services, including through retraining and continuing education programs for professionals in the field, and even developing specific job descriptions detailing the scope of work of palliative care providers. Another awaited development is the adoption of the National Procedures and Conditions of the Use of Narcotics/Psychotropic Substances for Medical Purposes, which will simplify the prescription of opioid medicines for severe pain by abolishing disproportionate requirements, including the need for police oversight of doctors prescribing opioids, biopsy-confirmed cancer diagnosis for the prescription of out-patient opioid pain medicine, multiple signatures on opioid prescription forms, and extensive record-keeping requirements imposed on physicians [40].

## Conclusion

Armenia’s efforts to integrate palliative care into its national healthcare system have contributed to strengthening human rights in patient care more broadly.

The framework of human rights in patient care clarifies state obligations and addresses the rights of both patients and providers. By applying human rights principles to the context of patient care, this framework looks beyond the individual patient-provider relationship to examine systemic issues and state responsibility. In the context of palliative care, these include patient rights to freedom from torture, cruel and inhuman treatment, non-discrimination and equality, bodily integrity, privacy and confidentiality, information, and right to a remedy. They also encompass provider rights to decent working conditions, freedom of association, and due process. The human rights in patient care framework enables a more holistic, balanced approach to healthcare services in general, including palliative care services in particular. As human rights apply to *everyone*, this framework acknowledges that providers do not solely have obligations but are also entitled to rights, which is essential to fostering a culture of respect for human rights within health care delivery systems [1].

In Armenia, the government’s efforts to integrate palliative care into health care, coupled with awareness raising and advocacy by human rights organizations, created an enabling environment for the realization of human rights in patient care. Armenia has adopted measures and is in the process of taking further ones to advance patient and provider rights, including strengthened laws, policies, guidelines, and training for specialized palliative care providers. These developments have, in turn, advanced human rights norms in patient care more broadly in Armenia.

## Abbreviations

CESCR: United Nations Committee on Economic Social and Cultural Rights
HRW Report: Human Rights Watch Report “All I Can Do Is Cry: Cancer and the Struggle for Palliative Care in Armenia”
ICESCR: International Covenant on Economic, Social and Cultural Rights and the International Covenant on Civil and Political Right
NGOs: Non-governmental organizations
WHO: World Health Organization
